# Research on PM_2.5_ concentration based on dissipative structure theory: a case study of Xi’an, China

**DOI:** 10.1038/s41598-020-73598-9

**Published:** 2020-10-02

**Authors:** Xiaoke Sun, Hong Chen, Zhizhen Liu, Hengrui Chen

**Affiliations:** College of Transportation Engineering, Chang’an University, Xi’an, 710064 China

**Keywords:** Environmental impact, Environmental impact, Environmental sciences, Environmental social sciences

## Abstract

PM_2.5_ pollution has become a serious urban environmental problem, especially in developing countries with increasing urbanization. Understanding the proportion of PM_2.5_ generation sources has laid a foundation for better PM_2.5_ concentration reduction This paper used Point of Interesting (POI)data, building profile data of Xi’an, PM_2.5_ concentration and wind monitoring data of five provinces near Xi’an as the basic data. And this paper studied the spatial distribution of various buildings in Xi’an, the temporal and spatial distribution of PM_2.5_ in Xi’an and the five provinces, and found that the spatial distribution of PM_2.5_ concentration in Xi’an and the building distribution in Xi’an does not match. Based on this, a quantitative model of PM_2.5_ concentration in Xi’an, energy consumption, wind, and other factors is established through the qualitative and quantitative analysis of PM_2.5_ concentration in Xi’an. Entropy theory and dissipative structure theory are applied to analyze this phenomenon. The results show PM_2.5_ in Xi’an mainly comes from the spread of PM_2.5_ in the five provinces. The PM_2.5_ generated by energy consumption in Xi’an is not enough to cause serious PM_2.5_ pollution. And further suggestions on how to reduce PM_2.5_ concentration in Xi’an are put forward.

## Introduction

With the rapid economic growth and development of infrastructure in developing countries, urbanization and motorization are accelerating. So are the demands in industry, living, and transportation based on fossil fuel, leading to serious urban air pollution^1^. PM_2.5_ refers to particles within an aerodynamic equivalent diameter of 2.5 microns in ambient air, which can levitate in the air for a long time. Although PM_2.5_ is only a fraction of the Earth’s atmosphere, it has a great effect on air quality and visibility. Its main chemical composition includes organic carbon, elemental carbon, nitrate, sulfate, ammonium salt, sodium salt^2^. With small particle size, it has strong activity, easily attached with toxic and harmful substances (such as heavy metal, microorganisms). Meanwhile, it has a long residence time and transportation distance in the atmosphere. It has great influence on health of human and air quality, reducing the overall comfort of the city^3^. In developing countries like China, with a rapid development in economy and infrastructure, there are many sources of PM_2.5_, causing large-scale environmental pollution. Therefore, it is very serious to find the primary factors of PM_2.5_ concentration to reduce it.

Under such circumstance, Chinese government spared no effort to improve urban transport system, the governance of industrial enterprises, and the transformation of domestic energy system (boiler transformation, bulk coal transformation), which greatly reduced PM_2.5_ emissions^4,5^. It can tell through the air pollution in lots of cities in China, especially in Beijing. In Beijing, one of the important measures to control urban air pollution is optimizing its energy structure. It has adopted stricter emission standards for coal-fired boiler pollutants. Financial subsidies are provided for replacement and reconstruction on coal-fired facilities. Meanwhile, it accelerates the development of clean energy such as natural gas and electricity. Comprehensive control of motor vehicle pollution is also carried out, including new and in-use vehicle control, promotion of new energy vehicles, improvement on quality of oil products, traffic control measures, economic incentives. An effective air quality management system has been established after such laws and regulations, effective law enforcement, systematic planning, strict local standards, sound monitoring systems, and rising public environmental awareness. These measures produced a very significant effect. During 2013 to 2017, the annual average concentration of PM_2.5_ dropped from 89.5 to 58 μg/m^3^.

With development of emerging geographic information systems (GIS), GPS technology, environmental detection technology, and big data mining methods, there are effective research methods for in-depth study of the sources of PM_2.5_ and analysis of the relationship between them^6–8^. With the big data mining method, we can analyze the real-time monitoring data of both PM_2.5_ and wind to find the correlation between them^9^. With GIS, the real-time monitoring data of PM_2.5_ and wind, and other interfering factors in each area can be visually displayed. It is useful to help researchers analyze their connection and identify related models with the spatiotemporal characteristics obtained from GIS^8,10^.

The famous physicist Prigogine introduced the concept of open systems to the second law of thermodynamics in 1969 and developed it to establish the dissipative structure theory. Dissipative structure theory explains how an open system changes from disorder to order, which is an extension of entropy theory. At present, dissipative structure theory has made a huge impact in many fields of natural sciences and social sciences, such as physics, astronomy, biology, economics, and philosophy^11^. However, research of dissipative structure theory on the diffusion of pollutants is rarely studied.

Some scholars have done some studies about Beijing. By omitting Traffic-related information like vehicle types, numbers and density of vehicles, they combined atmospheric chemical diffusion models with statistical GAMM models and simplified the mathematical algorithm. They found 17.2–37.3% of PM_2.5_ may be related to traffic emissions^12^. Some studies have proposed a comprehensive framework for estimating traffic-related air pollution concentrations in real-time traffic and basic meteorological information. The impact was further evaluated^13^. Adopting provincial panel data and panel data models from 2001 to 2012, some scholars analyzed the main driving forces affecting regional emissions at the regional level in China. It turns out the decisive factor of carbon emissions is economic growth. Carbon emissions vary from region to region, decreasing from central to western and eastern regions^14^. There are also studies analyzing the composition of collected PM_2.5_ by analyzing the chemical composition. But through this method, it is hard to distinguish whether the source of PM_2.5_ is in this region or not^3,15,16^. These studies considered factors such as energy consumption, vehicle emissions, economic growth and population growth without fully taking the impact of diffusion of pollutants from other surrounding cities^17–19^. Based on these studies, this paper adds the impact of peripheral urban pollutants on the research of urban pollutants.

It has been confirmed that the PM_2.5_ concentration is comprehensively affected by the factors such as energy consumption, economic growth, population growth, and vehicle emissions^20–23^. Nowadays, most researches on urban pollution focus on the analysis of pollution sources in a certain city, or large-region macro analysis of multiple cities. There are few studies that integrate these factors and the pollution of peripheral cities in the study area into a system for analysis. Therefore, these previous studies may be inaccurate. This study bases on the data of PM_2.5_ concentration and wind in five provinces (explained the province selection in “Regional elevation and terrain analysis of the five provinces” section) and data of influencing factors (such as energy consumption) in Xi’an to analyze the PM_2.5_ concentration in Xi’an. Taking advantage of a combination of qualitative and quantitative analysis methods, entropy theory, dissipative structure theory, this paper has studied the primary factors of PM_2.5_ concentration in Xi’an and analyzed how to reduce the PM_2.5_ concentration effectively. And this study will propose a more comprehensive PM_2.5_ reduction method than the previous one-sided and a more effective plan for improving the urban environment research on basis of a clear analysis of the source of PM_2.5_ in Xi’an and sources outside Xi’an.

The rest of this article is organized as followed: after this introduction, second section introduces materials and methods, including data and the theory; third section presents the case description; fourth section presents the results, discussions, and related implications; fifth section presents the conclusion.

## Materials and methods

### Data

This study aims to find the main source of PM_2.5_ pollutants in Xi’an. PM_2.5_ is mainly produced from industrial, living, economic, transportation and other aspects. As a class of tiny particulates, PM_2.5_ has a long residence time in the atmosphere and can spread a long distance with the wind. Therefore, whether PM_2.5_ in Xi’an has been increased by the diffusion of other areas should be considered. To analyze the relationship between PM_2.5_ concentration and wind, this study collected the PM_2.5_ concentration and wind speed of Xi’an (11 PM_2.5_ monitoring points) and five provinces (261 PM_2.5_ monitoring points and 45 wind monitoring points) from October 15, 2018 to April 15, 2019, according to the heating time in Northern China: November 15 to March 15, for a total of 183 days from China National Environmental Monitoring Station, China Meteorological Data Network, and National Centers for Environmental Information (https://www.ncdc.noaa.gov/). And the locations of PM_2.5_ monitoring points are divided into three categories: (1) Automatic monitoring points, located 3–15 m away from the ground; (2) Monitoring points located near buildings above 20 m, located 15–25 m away from the ground; (3) Roadside monitoring point is located 2–5 m away from the ground. It can be found in the National Environmental Protection Standard of the People’s Republic of China HJ 653-2013: Specification and Test Procedures for Ambient Air Quality Continuous Automated Monitoring System for PM_10_ and PM_2.5_. The monitoring data of wind direction is Land-Based Station Data, which is close to the ground level and can be found in National Centers for Environmental Information. Because the diffusion of PM_2.5_ in the urban area is greatly affected by the height and distribution of the building, this study obtained that data from Google Map to analyze its relationship. This study also collected the energy consumption (electricity, petroleum and coal), retail sales of consumer goods above designated size (Rs), consumption of automobiles and traffic income of Xi’an form National Bureau of Statistics and Xi’an Statistics Bureau to analyze the relationship between PM_2.5_ concentration of Xi’an and other factors.

### Spatial interpolation and General G

Spatial interpolation is a method of estimating unknown points by knowing points^24,25^. Limited by the number of environmental monitoring points, this study obtained air quality data from 11 monitoring stations in Xi’an and 261 monitoring stations in five provinces. This study attempts to analyze the temporal and spatial variation characteristics of Xi’an and five provinces air quality through spatial interpolation. In order to accurately simulate the spatial and temporal distribution of air quality in Xi’an and five provinces, the round model of the inverse distance weighting method, the spline method, the Kriging method, the spherical model, the exponential model and the Gaussian are respectively used. This study performs spatial interpolation and cross-validates the interpolation results. The optimal interpolation method is determined by comparing the mean absolute error (MAE) and the root mean square error (RMSE). The MAE and RMSE of the Kriging circular mode method are the smallest, which indicates that the method can reflect the spatial and temporal distribution pattern of Xi’an and five provinces air quality scientifically. Therefore, the circular function in Kriging method is used as the generation method of air quality surface data^24,25^.

General G is used to discriminate the spatial autocorrelation calculation and test of high-value aggregation and low-value aggregation of the POI data of Xi’an. The calculation formula is:

1 $$G=\frac{n{\sum }_{i=1}^{n}{\sum }_{j=1}^{n}W(i,j){X}_{i}{X}_{j}}{{\sum }_{i=1}^{n}{\sum }_{j=1}^{n}{X}_{i}{X}_{j}}$$

The large G value indicates a high value and a high value aggregation state in the unit area of the study area; the small G value indicates a low value and a low value aggregation state^25–27^.

### Entropy theory

Entropy comes from the Greek word for the capacity of change. It was coined by German physicist Clausius in 1856 to describe the second law of thermodynamics. As a state parameter, entropy is used to represent the uniformity of any kind of energy distribution in space.

In 1872, Boltzmann discovered the entropy in a single system is related to the number of microscopic states constituting the thermodynamic properties. Based on the study of statistical phenomena of molecular motion, the Boltzmann relationship was proposed:

2 $$S=K*lnN$$

The Boltzmann entropy *S* of the material system is equal to the product of the Bosch coefficient *K* and the logarithm of the number *N* of states.

The formula reflects the statistical significance of the entropy function. It links the macroscopic physical quantity S and the microscopic physical quantity N of the system, becoming one important bridge between macro and micro. Based on the relationship between the above entropy and thermodynamic probability, the following conclusions are obtained: The entropy value of the system directly reflects the degree of uniformity of the state it is in; The smaller the entropy value of the system, the more orderly and unevenly the state it is in; The larger the system’s entropy value, the more disordered and uniform it is; The system always tries to spontaneously change from a state with a small entropy value to a state with a large entropy value (from order to disorder).

As a state parameter, entropy is used to represent the uniformity of the distribution of many kinds of energy in space. However, in previous studies of pollutant diffusion, no scholar has used entropy to indicate the uniformity of the distribution of gaseous pollutants or small particulate pollutants in space. Pollutants can be considered as a kind of energy, and their diffusion can be regarded as the transfer of energy. In this research, the entropy value is creatively applied to the uniform distribution of PM_2.5_ in space to study the PM_2.5_ diffusion.

### Dissipative structure theory

An open system, far from the equilibrium state, constantly exchanges material energy with the outside world. When the external input system energy, material and information reach the threshold of the system’s self-organization, quantitative changes may cause qualitative changes. The entropy of the system is reduced, and the original disordered state is transformed into the ordered state. The ordered structure exchanges material, energy, and information with the external environment, being a necessary condition for the system to maintain vitality. Dissipative structure theory studies the nature of dissipative structures and the laws of their formation, stability, and evolution. It focuses on explaining how the open system moves from disorder to order. And it points out an open system far from the equilibrium state can continuously exchange materials and energy with the outside world. When the external conditions change to a certain threshold, it can generate self-organizing phenomena through internal action and make the system spontaneously change from the original disordered state to a macro-ordered state in space–time and function, forming a new and stable ordered structure. In this study, dissipative structure theory is used to analyze the source of pollutants in Xi’an. As Xi’an has met the four necessary conditions for a dissipative structure:

- Xi’an is an open system. With small volume and lightweight, PM_2.5_ is easy to spread along with the wind. Xi’an is not surrounded by mountains in four directions. From the perspective of PM_2.5_ diffusion, Xi’an is an open system.
- The PM_2.5_ system in Xi’an is far from equilibrium. The production and sales of consumer goods in Xi’an are unbalanced. Xi’an is in northern Shaanxi with flourishing tourism industry. However, the manufacturing industry cannot support the consumption brought by the tourism must, resulting in the imbalance between production and sales. There is also an imbalance between the occupation and living of urban residents. This imbalance results in a large amount of traffic. But demand and supply for the traffic are unbalanced in Xi’an, the urban traffic failed to meet the traffic demand. In addition, there is an imbalance between generation and diffusion of PM_2.5_ in winter in Xi’an. Due to the low temperature in winter, centralized heating and energy consumption caused a large amount of PM_2.5_. However, without strong wind or much rain and snow, it is not conducive for PM_2.5_ to diffuse.
- The PM_2.5_ concentration in Xi’an is a non-linear interaction. As mentioned above, PM_2.5_ is generated by various methods such as industry, energy, life, economy, transportation. As a very important factor to affect the concentration, the diffusion is affected by weather factors such as wind. Therefore, PM_2.5_ concentration is formed not under a simple linear interaction, but under a very complex interaction.
- There are random huge fluctuations in the PM_2.5_ system in Xi’an. Any cause of generation and spread of PM_2.5_ may cause a sudden increase or decrease in PM_2.5_ concentration. For example, a sudden increase in wind speed would increase the spread of PM_2.5_, leading to a decrease in PM_2.5_ concentration. A sudden increase in the number of tourists on a holiday would result in an increase in traffic and a rise in PM_2.5_ concentration.

When the system exchanges energy with another system, there must be an energy difference between the two systems. Due to the difference, energy would spontaneously flow from the system with high energy to the system with low energy. Entropy can represent the uniformity of the material distribution of a system in the environment. If the entropy of a system is larger, the state is more disordered, indicating the entropy in the system is more uniform. And if the material is completely uniformly distributed, then the entropy of this system has reached its maximum value. Considering Xi’an as a system, the entropy represents the concentration of PM_2.5_. Xi’an has typical dissipative structural properties, and is a high-level system with human participation as the central subject.

## Case description

Xi’an is a famous tourist city and 13 dynasties established capital in Xi’an. It has a total area of 10,752 square kilometers. In this study, the main urban area of Xi’an, including Weiyang District, Baqiao District, Lianhu District, Beilin District, Xincheng District and Yanta District, was mainly discussed. At the end of 2018, the total resident population of the main urban area of Xi’an was 4.8875 million, increasing 0.4 million compared to 2010. Annual GDP was 564.022 billion yuan, increasing 340.31 billion yuan compared to 2010. Electricity consumption of the whole city of Xi’an increased from 1.99 to 3.96 million kilowatt-hours, exacerbating social energy consumption. And the number of vehicles was 3.329 million in 2018, increasing 2 million compared with 2010. It not only indicates the rapid economic growth of Xi’an, but also problems such as the pollution.

**Figure 1:**
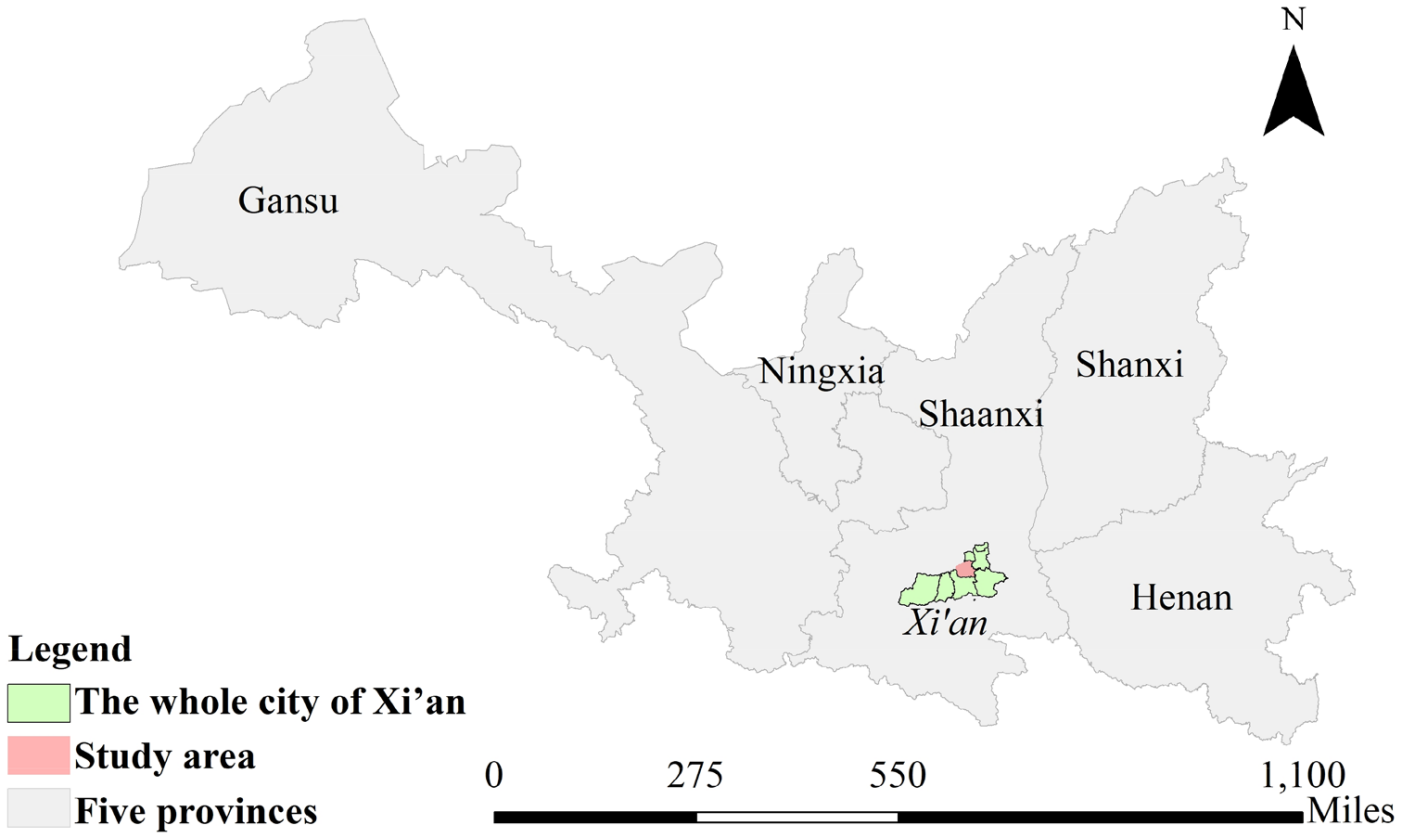
Study area. Plotted the main urban area of Xi’an (study area), including Weiyang District, Baqiao District, Lianhu District, Beilin District, Xincheng District and Yanta District, and other area of Xi’an and five provinces. And figure was plotted by ArcGIS 10.6.0.8321, which can be downloaded on https://www.esri.com.

Xi’an is located south of the Guanzhong Plain and the middle of the Yellow River Basin, the geographic center of China, having a warm temperate semi-humid continental monsoon climate. The winter is cold, light wind, foggy, less rain and snow. It is extremely unfavorable to the diffusion of PM_2.5_, but very easy to produce PM_2.5_. The study area is shown in Fig. 1.

## Result and discussion

### Analysis of influencing factors of PM_2.5_ in Xi’an

**Figure 2:**
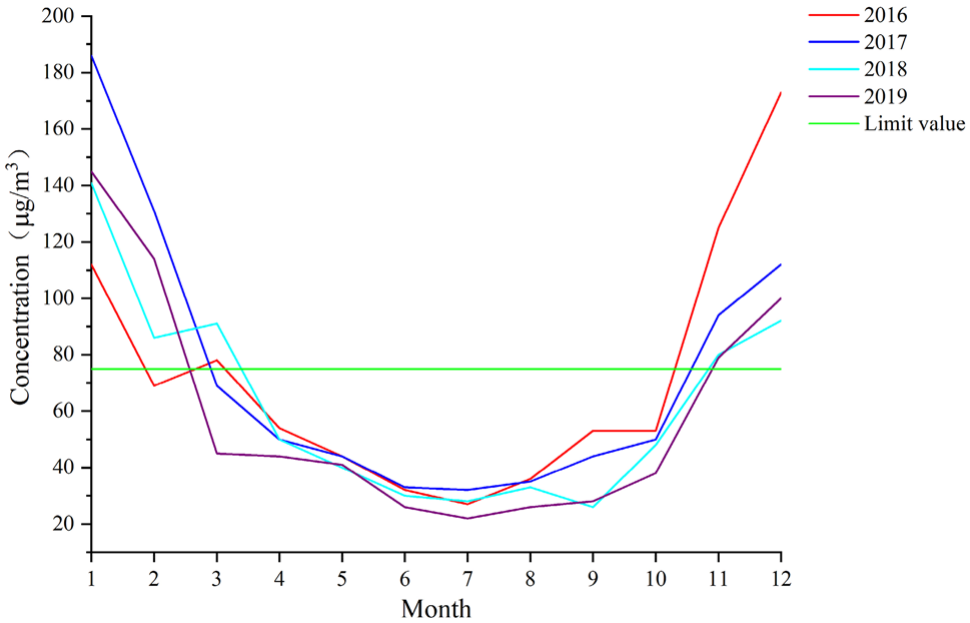
PM_2.5_ concentration by month in 2016–2019. The average monthly PM_2.5_ concentration in 2016–2019 is compared with the limit value. And figure was plotted by OriginPro 2020b (64-bit) 9.7.5.184 (Student Version), which can be downloaded on https://www.originlab.com.

According to the air quality standard issued by China, the limit of PM_2.5_ concentration is 75 μg/m^3^. The average monthly PM_2.5_ concentration in 2016–2019 is compared with the limit value, showing in Fig. 2. It fell below the limit in March to April and rose above the limit in October to November, showing a V-shaped distribution with the lowest in July and the maximum in January or December. Xi’an has severe pollution in winter and light pollution in summer, as other cities in China.

**Figure 3:**
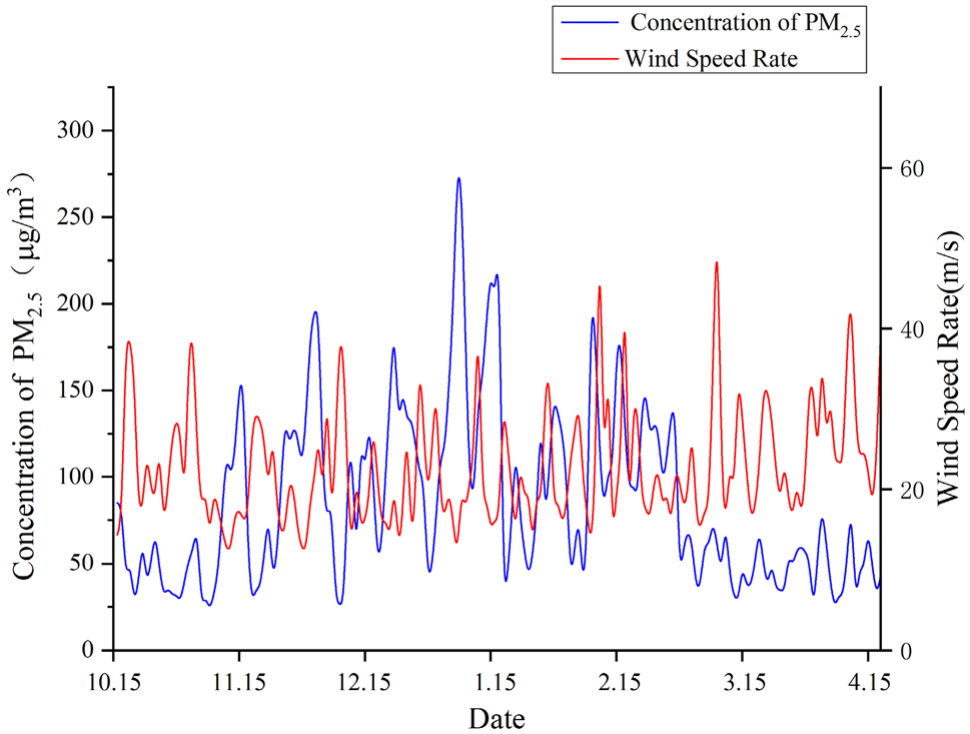
PM_2.5_ concentration and wind speed by day. The daily PM_2.5_ concentration and wind speed at corresponding time in Xi’an. And figure was plotted by OriginPro 2020b (64-bit) 9.7.5.184 (Student Version), which can be downloaded on https://www.originlab.com.

The daily PM_2.5_ concentration and wind speed at corresponding time in Xi’an was plotted in Fig. 3. The correlation coefficient between them is − 0.26757, being weak negative. The PM_2.5_ concentration is related to the wind speed, but many factors such as vehicle, life and industry would be influential.

**Figure 4:**
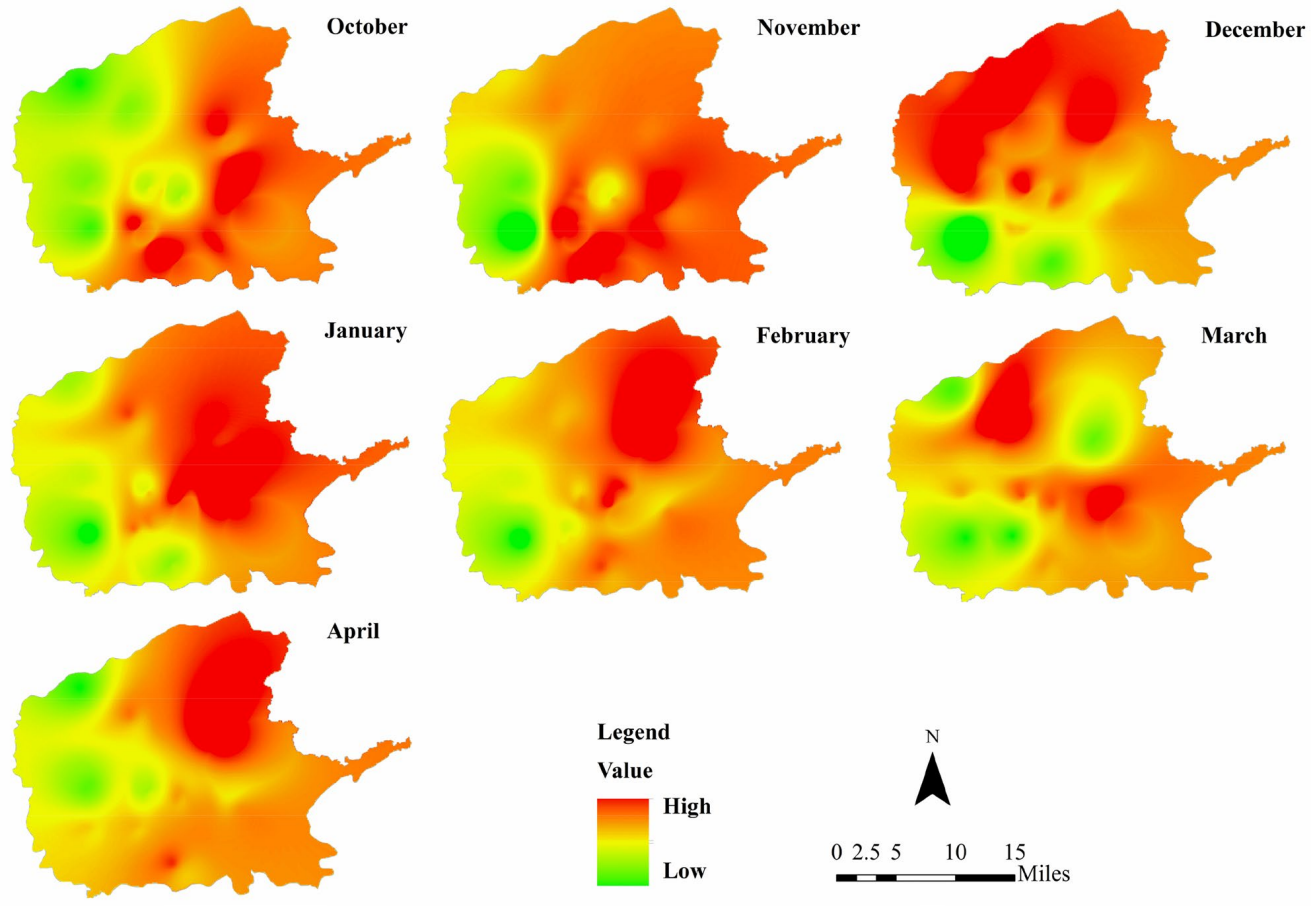
PM_2.5_ concentration distribution from October 2018 to April 2019. PM_2.5_ concentration distribution from October 2018 to April 2019 was plotted in figure. And figure was plotted by ArcGIS 10.6.0.8321, which can be downloaded on https://www.esri.com.

The PM_2.5_ in Xi’an is mainly distributed in the east and north, showing in Fig. 4. Urban spatial structure has great impact on the generation and spread of PM_2.5_. Such as the imbalance of occupational and residential areas would increase traffic, the development of public transportation in the city would have a great impact on using private car, and the height of urban buildings has great impact on the diffusion of PM_2.5_.

**Figure 5:**
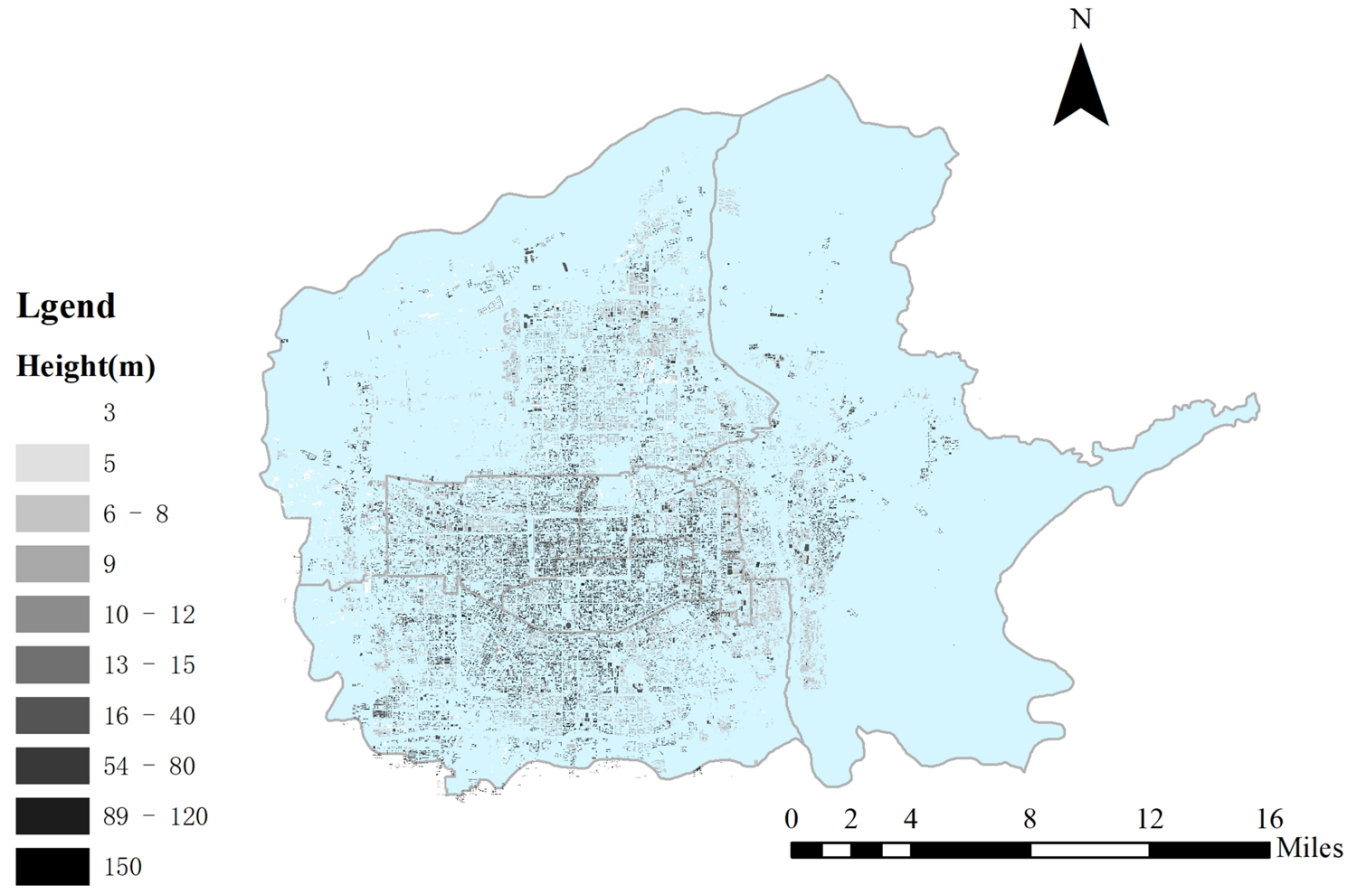
Building distribution in Xi’an. Xi’an building height distribution map is plotted in figure. And figure was plotted by ArcGIS 10.6.0.8321, which can be downloaded on https://www.esri.com.

Xi’an building height distribution map is plotted in Fig. 5, showing the tall buildings in Xi’an are mainly located in the central and southern part of the city, especially within the second ring road of Xi’an, and the distribution of high-rise buildings around Xi’an is relatively scattered. Therefore, Xi’an is a sprawling city with high-rise buildings concentrated in urban center and radiating to the surroundings. As population growth of sprawling city could not keep up with its land growth, excessive and rapid development of land brings waste of natural resources. Sprawling structure would reduce land use efficiency, increase energy consumption and PM_2.5_.

**Figure 6:**
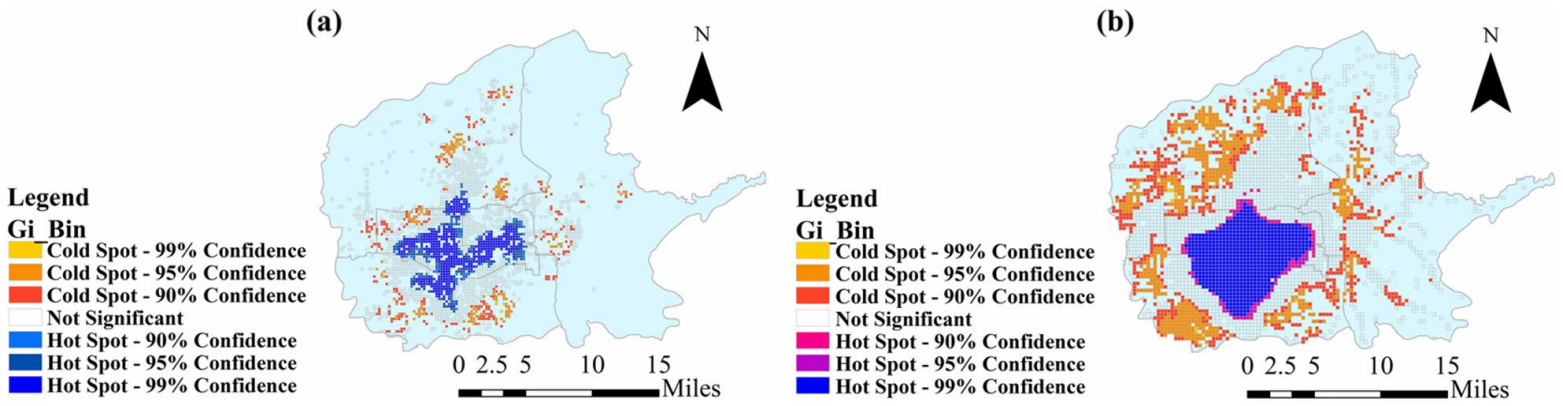
Cold and hot spot analysis of residential area (**a**) and other area (**b**). Figure is the cold and hot spot analysis of POI points of residential area (**a**) and other areas except residential (**b**) in 6 districts (Yanta, Beilin, Weiyang, Baqiao, Xincheng, and Lianhu) in Xi’an. And figure was plotted by ArcGIS 10.6.0.8321, which can be downloaded on https://www.esri.com.

Figure 6 is the cold and hot spot analysis of POI points of residential area (a) and other areas except residential (b) in 6 districts (Yanta, Beilin, Weiyang, Baqiao, Xincheng, and Lianhu) in Xi’an. It shows that the clustering of residential area does not match the clustering of other area. The area of urban residents is inconsistent with areas for work, shopping and entertainment, increasing activities with long distance and number of travelers.

**Figure 7:**
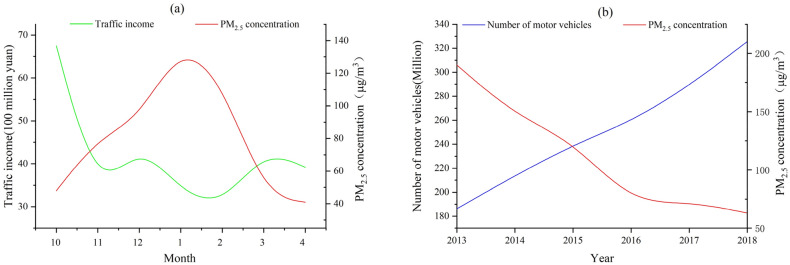
PM_2.5_ concentration and traffic. Figure maps the traffic income and the number of vehicles in Xi’an with the average PM_2.5_ concentration. And figure was plotted by OriginPro 2020b (64-bit) 9.7.5.184 (Student Version), which can be downloaded on https://www.originlab.com.

Motor vehicle transportation contributing much to the generation of PM_2.5_, but it could not determine the final concentration, which is determined by various factors. Figure 7a maps the traffic income in Xi’an and the average PM_2.5_ concentration from October 2018 to April 2019 in each month, showing there is no obvious positive linear relationship. Figure 7b maps the number of vehicles and the annual average PM_2.5_ value from 2013 to 2018. The number of vehicles is increasing year by year, which is closely related to the improvement of people’s life quality in Xi’an. However, Fig. 7b shows the annual average concentration of PM_2.5_ is decreasing, which indicates that the number of vehicles cannot determine the eventual concentration of PM_2.5_.

The high PM_2.5_ concentration in the north of Xi’an does not match the urban distribution of Xi’an. From Figs. 5 and 6, it can be seen main urban area of Xi’an is in the central and southern parts and so the residential area. Although the working and entertainment areas are denser than the residential area in northern, they are still relatively sparse compared to the central and southern. Therefore, the northern part of Xi’an belongs to an area that has not been fully developed. It has a high PM_2.5_ concentration, indicating that the PM_2.5_ concentration in Xi’an is not only related to the energy consumption, building distribution, and economic development in Xi’an, but also has a strong relationship with the PM_2.5_ diffusion outside Xi’an.

### Spatial analysis of PM_2.5_ in Xi’an and its surroundings

#### Regional elevation and terrain analysis of the five provinces

**Figure 8:**
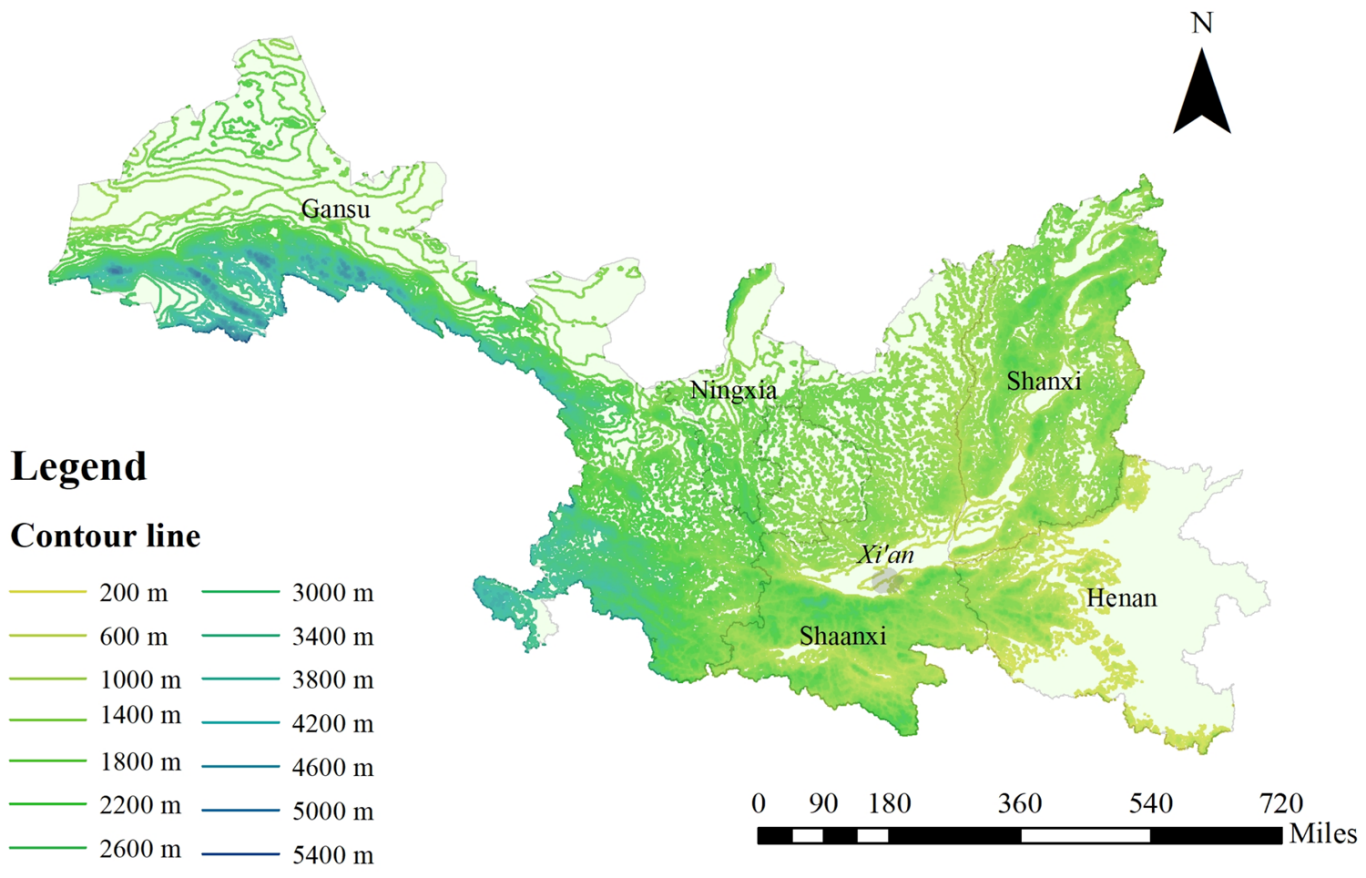
Contour line of five provinces. Figure mapped the contour line of five provinces. And figure was plotted by ArcGIS 10.6.0.8321, which can be downloaded on https://www.esri.com.

As can be seen from the Fig. 8, Xi’an is in the south-central of the five provinces, the central part of the Guanzhong Plain. Longitude is 107.40° to 109.49° east and 33.42° to 34.45° north latitude. It is adjacent to the Qinling Mountains, and the north is the main component of the Guanzhong Plain. There are no mountains in the northeast to pass through Weinan city, Yuncheng city and Linfen city to reach Taiyuan city. The west is bounded by Taibai Mountain and Qinghua Loess Terrace. It passes through Weinan city to the east and is adjacent to Henan and Shanxi province. Xi’an has the highest altitude difference among cities in China. In the south of Xi’an, wind is difficult to pass due to the barriers of the Qinling Mountains. Therefore, the diffusion of PM_2.5_ effects in the three regions of Sichuan, Hubei province and Chongqing city is not considered. Shanxi and Henan provinces have lower elevations and fewer mountains, which are conducive to the diffusion of PM_2.5_, and they are also two highly polluted provinces in China. Gansu Province and Ningxia Hui Autonomous Region are close to Shaanxi Province and located in the north of Xi’an with no high mountain barrier, having an important impact on the diffusion of PM_2.5_. Therefore, these five provinces were selected to analyze the diffusion of PM_2.5_.

#### Analysis of the impact of five provinces on PM_2.5_ concentration in Xi’an

**Figure 9:**
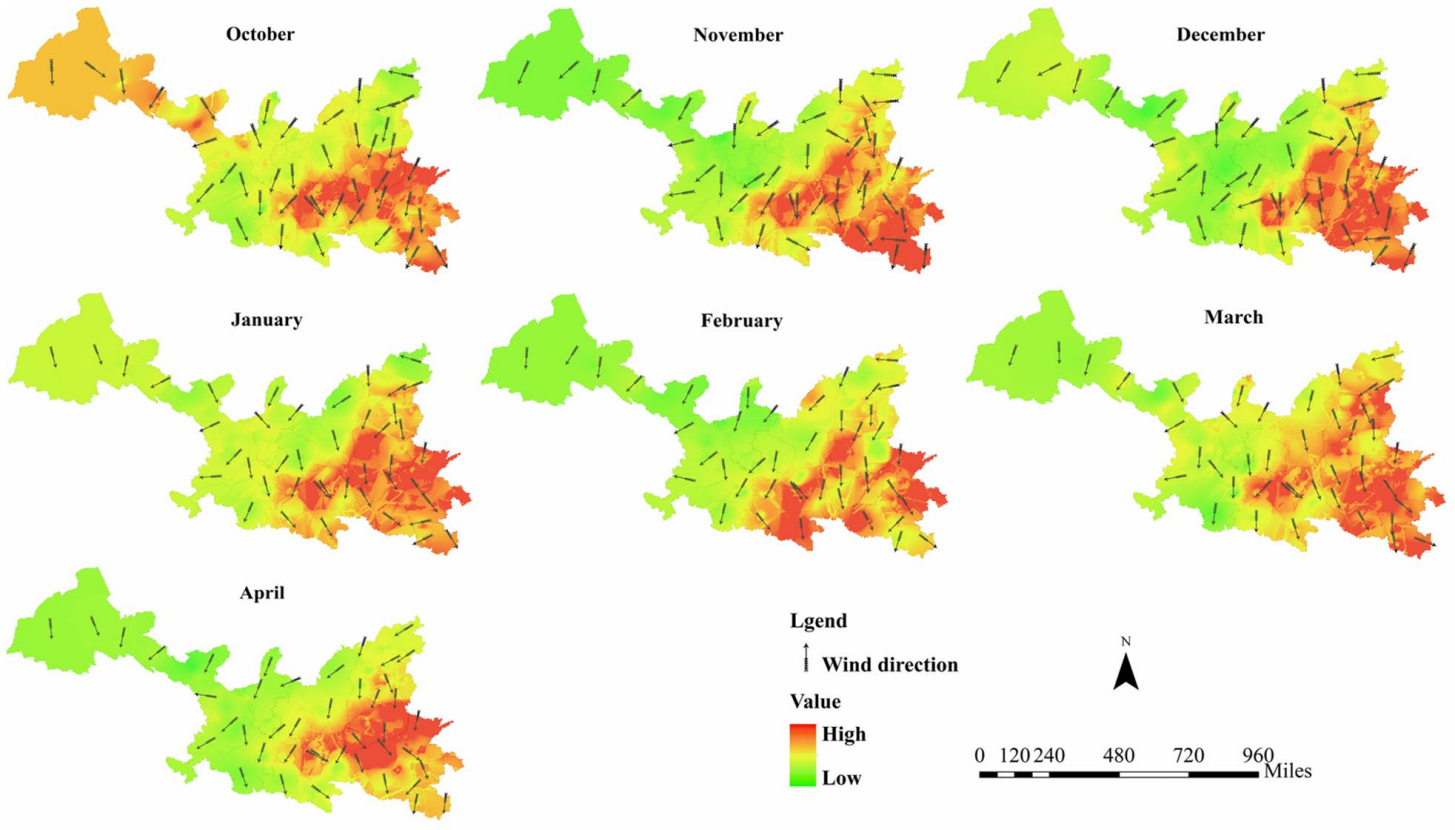
The max PM_2.5_ concentration and wind direction distribution. The distribution of PM_2.5_ concentration and the average wind direction of the five provinces in 15 days matching the date of the max PM_2.5_ was mapped in figure. And figure was plotted by ArcGIS 10.6.0.8321, which can be downloaded on https://www.esri.com.

The diffusion of PM_2.5_ is a time-consuming process. For example, when the PM_2.5_ concentration in Xi’an is lower than other areas, the outside PM_2.5_ slowly spreads to Xi’an, resulting in the increase. The entire diffusion process takes some time. When considering the impact of other provinces on Xi’an, the difference between the dates of the maximum and minimum PM_2.5_ concentrations in Xi’an within a month is calculated, and the average is used as a diffusion time to balance the effect of time on the error caused by diffusion. The final is “15 days”, showing in Table 1. The distribution of PM_2.5_ concentration and the average wind direction of the five provinces in 15 days matching the date of the max PM_2.5_ was mapped in Fig. 9. When calculating the wind direction of 45 wind monitoring points in October, for example, this study calculated the average wind direction of 10.01–10.15 (15 days before Date of the max PM_2.5_).The heavily polluted areas are in the east and south of the five provinces: Henan, Shaanxi, and Shanxi, and the wind direction is mainly northerly. For example, in March, the wind direction is like a C-shaped arc: the main wind in the north of Shanxi is the northeast wind, which changes to the northwest wind in the Middle Shanxi. The northwest wind blows across Shaanxi and Henan as the main wind. Therefore, a large part of pollutants in Shanxi would spread to Shaanxi and Henan along with the wind. There is lower PM_2.5_ concentration in Ningxia Hui Autonomous Region and Gansu, and the wind in these two provinces is mostly southeast. The pollutants in these two provinces are not easy to spread to Shaanxi Province and have a small effect on the pollutant concentration.

**Table 1:** Diffusion time calculation.

Date of the max PM_2.5_	Date of the min PM_2.5_	Difference	Average
10.16	10.19	3	15
11.30	11.06	24	
12.01	12.07	6	
1.05	1.15	10	
2.12	2.26	14	
3.09	3.30	21	
4.12	4.04	8

### Qualitative and quantitative analysis of PM_2.5_ in Xi’an

#### Qualitative analysis

Considering Xi’an as a system, factors affecting PM_2.5_ concentration are mainly divided into two categories: PM_2.5_ generated in Xi’an and PM_2.5_ spread to Xi’an.

In the north of China, heating will be supplied from mid-November to mid-March. It requires a large amount of energy consumption from coal, gas, oil, and electricity, which would produce a lot of PM_2.5_.

Vehicle emission is an important source of PM_2.5_ and a lot of pollutants are generated from the burning of fossil fuels in vehicles. At present, it is inevitable for vehicle emissions. As public transportation is only a part of the urban traffic and there are few electric vehicles. Much more urban traffic is necessary for urban residents working, schooling and entertainment. Therefore, vehicle emission is still a major source of PM_2.5_.

The PM_2.5_ is mainly generated from the material transportation, material handling and earth excavation in the building construction. Besides, laying pipe networks, handling materials such as cement and white ash will also produce PM_2.5_. Generally, there is an inverse proportional relationship between the humidity and the amount of dust from the construction site. The dust from the construction site would have a serious effect on the surrounding environment with a strong wind or dry weather. Therefore, PM_2.5_ concentration can also be affected by the construction. But the effect is limited according to the requirements of urban environmental protection. The construction should be enclosed when operation.

Industry would produce pollutants like sulfur oxides, nitrogen oxides, and PM_2.5_. PM_2.5_ is mainly smoke and dust emitted from the combustion of various combustibles. In addition, it can also be converted by sulfur and nitrogen oxides.

Xi’an is in the center of Shaanxi Province. Qinling Mountains are in the southern part of Xi’an, which stops the spread of PM_2.5_. From the distribution maps of PM_2.5_ concentration and wind direction, Shanxi, Shaanxi and Henan, located in the east and south of the whole region, are the most seriously polluted by PM_2.5_ in the five provinces. When the concentration of PM_2.5_ in Xi’an was the largest, the wind in the five provinces was mostly northerly. In the north of Shanxi Province, the main wind was from northeast and changed to northwest wind in the Middle Shanxi. The northwest wind blew across Shaanxi and Henan as the main wind, which eventually presented a C-shaped arc. Along with the wind, much PM_2.5_ in Shanxi would spread to Shaanxi and Henan. Therefore, it should be considered that the diffusion of PM_2.5_ from external system has influence on PM_2.5_ concentration in Xi’an.

#### Quantitative analysis

Firstly, energy, transportation, economy, and wind were selected as influencing factors of PM_2.5_ concentration in Xi’an, and PM_2.5_ concentration and wind in five provinces were modeled as influencing factors outside Xi’an:

3 $${PM}_{2.5}\left(X{i}{\text{'}}an\right)=Xi{\text{'}}an +FP +{\mathrm{h}}N$$

4 $$Xi{\text{'}}an =Energy\left({E}_{c} + {C}_{c} + {P}_{c}\right)+{\mathrm{ f}}W + Ttraffic \left({T}_{ti} + {T}_{ac}\right)+ {jC}_{l}$$

5 $$FP={\mathrm{ g}}\sum {PM}_{2.5}* {W}_{s} * COS\left(\alpha -\theta \right)/L$$

where *PM*_2.5_(*Xi’an*) is the PM_2.5_ concentration in Xi’an, *Xi’an* is the affecting factors of PM_2.5_ concentration in Xi’an, *FP* (five provinces) is the affecting factors of PM_2.5_ concentration outside Xi’an, and $N$ is other affecting factors.${E}_{c}$ is electricity consumption, ${C}_{c}$ is coal consumption, and ${P}_{c}$ is petroleum consumption. *W* is wind speed in Xi’an. ${T}_{ti}$ is traffic income, and ${T}_{ac}$ is automobile consumption. ${C}_{l}$ is limit Rs. ${PM}_{2.5}$ is the PM_2.5_ concentration in the monitoring points, ${W}_{s}$ is wind speed in the monitoring points, $\alpha$ is the angle between the ray from the monitoring point to Xi’an and the ray from the north direction of the monitoring point, $\theta$ is the wind direction angle, and $L$ is the distance from the monitoring point to Xi’an. After fitting with the relevant data in the model, the adjusted R-squared is 0.97742.

Then, energy and wind were selected as influencing factors of PM_2.5_ concentration in Xi’an, and PM_2.5_ concentration and wind in five provinces were modeled as influencing factors outside Xi’an:

3 $${PM}_{2.5}\left(Xi{\text{'}}an\right)=Xi{\text{'}}an +FP +{\mathrm{ h}}N$$

6 $$Xi{\text{'}}an =Energy\left({E}_{c} + {C}_{c} + {P}_{c}\right)+{\mathrm{ f}}W$$

5 $$FP={\mathrm{ g}}\sum {PM}_{2.5}* {W}_{s} * COS\left(\alpha -\theta \right)/L$$

After fitting with the relevant data in the model, the adjusted R-squared is 0.99384.

The first model selects electricity, coal, petroleum, wind, transportation income, automobile consumption, Rs as the affecting factors in Xi’an and PM_2.5_ concentration, wind, distance as affecting factors in the five provinces. The PM_2.5_ generated by transportation and Rs in the first model is basically due to energy consumption. Firstly, transportation is generated by vehicle fuel. Secondly, PM_2.5_ of Rs is generated by economic growth, which is caused by the rapid development of industries such as manufacturing and transportation, and cannot be separated from energy consumption. Therefore, this study attributes economic and transportation to energy consumption. And the second model has a higher degree of fitting, so the second model is selected in this study.

The fitted model is:

7 $${PM}_{2.5}\left(Xi{\text{'}}an\right)={\mathrm{a}}{E}_{c}+{\mathrm{s}}{C}_{c}+{\mathrm{d}}{P}_{c}+{\mathrm{f}}W+{\mathrm{g}}\sum {PM}_{2.5}*{W}_{s}*COS\left(\alpha -\theta \right)/L+{\mathrm{h}}N$$

The parameters are in Table 2.

**Table 2:** Parameters of the fitted modle.

Parameter	value	Parameter	Value
a	2.01545	f	0.09238
s	70.79386	g	510.05844
d	0.29658	h	− 123.78923

### Analysis of dissipative structure

This study defines pollution entropy as another form of pollutant concentration. The larger the pollution entropy, the higher the pollutant concentration, the worse the environment, and the more unstable the system.

8 $${Si}_{e}=-\sum_{ie}^{n}{K}_{ie}{P}_{ie}ln{P}_{ie}$$

9 $${Si}_{q}=-\sum_{iq}^{n}{K}_{iq}{P}_{iq}ln{P}_{iq}$$

10 $$So=-\sum_{o}^{n}{K}_{o}{P}_{o}ln{P}_{o}$$

11 $$S\left(Xi{\text{'}}an\right)={S}_{i}+{S}_{o}$$

where ${Si}_{e}$ is the entropy generated by energy consumption, ${Si}_{q}$ is the entropy generated by other factors, and $So$ is the entropy generated by the outside system. ${S}_{i}$ is the increase of entropy generated by system’s own irreversible process, which can only be positive. ${S}_{o}$ is the negative entropy flow caused by interdiffusion with the outside system, which can be positive, negative, or zero.

If *S*(*Xi’an*) is zero and ${S}_{i}$ is equal to $-{S}_{o}$, the system is in a steady state. If *S*(*Xi’an*) is greater than zero, the negative entropy flow is not enough to offset the entropy generated by the system (including the positive entropy flow supplied by the outside system). The system is in an unstable state. And the system structure needs to be improved to reduce the potential risk energy. If *S*(*Xi’an*) is less than zero, the negative entropy flow is enough and the system is in a benign state, developing in a more orderly way. The dissipative structure is in a non-linear interaction area far from the equilibrium. When the tissue is disturbed, it will be amplified by the system. When it reaches the critical value region, it will cause huge fluctuations. This study analyzed the valve area where the huge fluctuation would occur based on the characteristics of the dissipative structure. It will help make good decisions in a right time and evaluate the effectiveness of decisions. Calculate the entropy of Xi’an in Table 3.

**Table 3:** Entropy from October to April.

Month	${Si}_{e}$	${Si}_{q}$	*So*	*S*(*Xi’an*)	*PM*_2.5_(*Xi’an*)
10	18.41003	− 34.3859	109.7717	93.79578	48
11	20.39311	− 34.3879	156.4123	142.4175	80
12	19.77613	− 34.3867	156.3481	141.7375	92
1	19.5134	− 34.3876	166.8659	151.9916	145
2	21.17256	− 34.3861	165.4693	152.2558	114
3	22.29282	− 34.3856	98.15763	86.06481	45
4	20.11987	− 34.3836	94.74269	80.47899	44

Using ${PM}_{2.5}\left(Xi{\text{'}}an\right)$ and $S\left(Xi{\text{'}}an\right)$ to do a linear fit:

12 $${PM}_{2.5}\left(Xi{\text{'}}an\right)=-48.55278+1.06966*S\left(Xi{\text{'}}an\right)$$

Table 4 is the proportion of energy entropy, external entropy, and other entropy in each month of Xi’an. Energy entropy, as the important source of positive entropy flow in Xi’an, accounts for 8–15% of the total. External entropy, as the main source of positive entropy flow in Xi’an, accounts for 63–76% of the total entropy. Other entropy, including the wind, as the main negative entropy source, accounts for 15–24% of the total. ${Si}_{q}$ is not enough to offset the positive entropy, so the total entropy of Xi’an shows a state of *S*(*Xi’an*) greater than zero. In order to offset the positive entropy flow, it is necessary to start with the generation source. When *PM*_2.5_(*Xi’an*) is equal to 75, the national concentration limit, *S*(*Xi’an*) is equal to 115.506591. Therefore, 115.506591 is the threshold of the entropy. When the dissipative structure reaches the threshold, a self-organizing phenomenon can be generated by the internal action, so that the system spontaneously changes from the original disordered state to the macroscopic ordered state in space–time and function, forming a new and stable ordered structure. When Xi’an reaches the PM_2.5_ concentration threshold, government departments and citizens would self-organize and proactively propose various solutions to reduce the PM_2.5_ concentration.

**Table 4:** Entropy ratio from October April.

Month	${Si}_{e}$	${Si}_{q}$	*So*
10	0.113245	0.211518	0.675237
11	0.096561	0.162827	0.740612
12	0.093943	0.163349	0.742708
1	0.088389	0.155764	0.755846
2	0.095791	0.155573	0.748635
3	0.143977	0.222078	0.633945
4	0.13481	0.230382	0.634808

The combustion of coal and oil is an important source of positive entropy flow. Especially in the heating period, the positive entropy flow generated by the combustion of coal and oil is higher than October and April. Since heating is a necessary condition for life, the only way to reduce the entropy is to improve heating efficiency and find alternatives.

Both industry and construction need large amount of energy. To reduce the energy consumption of them, regulations on energy consumption and strict requirements on pollution emissions must be formulated, such as relevant enterprises are required to carry out the treatment of pollutants before the pollutants discharged to meet the corresponding pollution emission standards^28^.

To reduce energy consumption due to transportation, many measures have been made in China.

- Promote the priority development of public transportation. Public transportation is an important way for urban residents. The current development of public transportation is not very good in Xi’an. Buses cannot carry a corresponding number of commuting passengers at a specific time, causing a sudden surge in commuting traffic and congestion during commuting hours, which lead to the increase of energy consumption during driving. Idle driving is with the lowest efficiency of vehicle driving, but congestion has caused much vehicles idling. Congestion also increases the time for energy consumption. Due to the special geographical environment, there are only four subway lines in Xi’an. Xi’an is an ancient capital for 13 dynasties, and there are many cultural relics underground. Under the conditions of protection of these cultural relics and the optimization of the line, the construction of the subway is very slow, leading to insufficient sharing of the public transport and an increase in private car travel. At the same time, the increase in population will also bring about changes in traffic. Public transportation facilities set by the government at the beginning may not keep up with the development, leading to an increase in cars and emissions. To solve the current problems of public transportation, some measures should be implemented. Improve the distribution of bus stations to meet the commuting demands of urban residents and avoid private car travel due to commuting demands. Reasonably dispatch buses and try to meet the demands of commuting at the peak time of commuting. Improve distribution of occupational and residential areas to reduce the traffic due to the imbalance between them.
- Promote green energy^29,30^. The government advocates vehicles with new energy, such as natural gas and electricity. At present, buses and taxis have completed the transition from fuel to new energy in Xi’an. Some private cars have used electricity and natural gas as power, but there are still many private cars are powered by fuel. It will be a long process of three reasons to improve the power of the vehicle. First, it is difficult to recycle private fuel cars. Most urban residents own private cars, having a specific service life. It is a financial problem for residents to change their private fuel cars to new energy vehicles within a short time. Second, compared with fuel vehicles, there are many problems in driving with new energy vehicles. The power is insufficient and the driving experience is not as good as fuel vehicles. Many drivers who are used to driving fuel vehicles cannot adapt the new energy vehicles for the first time. And because of insufficient power, new energy vehicles cannot provide corresponding services for the drivers, such as rapid climbing, high-speed driving. Finally, how to charge electric vehicles remains a problem. With unreasonable setting of charging pile, the electric vehicle cannot be charged in time, resulting in insufficient electricity during driving, which is unable to meet the demands. And if the electric vehicle is out of power during driving, it does take some time to charge. Here are some suggestions to help solve the current problems of new energy vehicles. Encourage urban residents to use new energy vehicles, and provide corresponding subsidies. Improve the charging facilities for electric vehicle as soon as possible. Improve the charging time, so as not to waste time when charging. Set up special charging stations and prepare backup batteries. When the electric vehicle power is less than 10%, the driver can go to the charging station to replace the battery, and the replaced battery can be charged in the charging station. The problem of insufficient power of the electric vehicle is needed to be solved as soon as possible for the problems in climbing and speeding due to insufficient power.
- Reasonable restrictions should be carried out. At present, daily traffic restrictions are implemented in Xi’an. The vehicle is restricted by the tail number (if the tail number is an English letter, the last digit of the license plate would be taken) of the license plate (including temporary license plate). The tail number of the vehicle restricted is 1 and 6 on Monday, 2 and 7 on Tuesday, 3 and 8 on Wednesday, 4 and 9 on Thursday and 5 and 0 on Friday. After restriction, the amount of traffic has been effectively reduced in Xi’an. However, due to the large number of vehicles in Xi’an, traffic congestion still exists. After the implementation of the traffic restriction, many residents with good incomes choose to purchase another motor vehicle to meet their daily travel demand. And it requires the government department to implement the purchasing restriction.

Negative entropy flow is mainly provided by the wind inside Xi’an, but wind is an uncontrollable factor in nature. Therefore, it is hard to increase the negative entropy flow from the wind. In addition, due to the effective use of policies, traffic organization, it would reduce the generation of positive entropy flow in Xi’an, so it is also an increase of negative entropy flow.

The positive entropy flow in Xi’an is mainly provided by the outside, affected by the wind. To fundamentally reduce this type of positive entropy flow, reducing the PM_2.5_ concentration outside Xi’an is important, which is like the method mentioned above. However, due to the different industrial structures of different provinces and cities, the method of reducing energy entropy generation is also different, so it should be determined according to the actual situation of each province and city.

Many other studies have used different models to analyze the source of PM_2.5_ in a city, but there are few ones considering both factors inside and outside the city. And there are also studies analyzing the composition of collected PM_2.5_ by analyzing the chemical composition. But through this method, it is hard to distinguish whether the source of PM_2.5_ is in this region or not. This study uses the dissipative structure theory to synthesize the influencing factors inside and outside Xi’an, and gives a more detailed analysis method.

## Conclusions

This paper has studied the relation between PM_2.5_ concentration in Xi’an and the energy consumption wind, economy, and transportation in Xi’an, as well as to wind and PM_2.5_ concentration from the five provinces around Xi’an from October 2018 to April 2019. It was found that the PM_2.5_ concentration in Xi’an was mainly related to the diffusion of the PM_2.5_ in the five provinces. The main source of PM_2.5_ in Xi’an is the energy consumption. And the main factor of PM_2.5_ reduction is the wind power in Xi’an. This study takes PM_2.5_ as the result of the increase or decrease of the entropy of Xi’an using the theory of dissipative structure to build the PM_2.5_ dissipative structure. The results show that energy entropy is the main source of positive entropy flow in Xi’an, accounting for 8–15%. As the main source of positive entropy of the total entropy of Xi’an, the external entropy affects the size of the total entropy, accounting for 63–76%. Other Entropy, including the wind and other factors in Xi’an, is the main source of negative entropy flow total entropy of Xi’an, accounting for 15–24%, which cannot offset the positive entropy flow generated in Xi’an and the positive entropy flow brought by the out system. Therefore, the total entropy of Xi’an shows a state of $S\left(Xi{\text{'}}an\right)$ greater than zero. Since less negative entropy flow in Xi’an, reducing the source of positive entropy flow should be taken to improve the environment and decrease the PM_2.5_ concentration in Xi’an. Heating is a necessary for the life of urban residents. Therefore, the only way to reduce the positive entropy flow due to heating is to improve heating efficiency and find alternatives. In terms of energy consumption from industry and building, the government should formulate corresponding provision on energy consumption and implement strict requirements on pollution emissions, requiring relevant enterprises to perform pollutant treatment to meet the pollution emission standards. For transportation, public transportation and green energy should be developed first and reasonable restrictions should be carried out on traffic. From the perspective of reducing the PM_2.5_ concentration in the cities and provinces around Xi’an, the method is like the method of reducing the generation of positive entropy mentioned above. But the method is different in each city due to the different industrial structures, which should be taken according to the situation of each city. Therefore, in order to reduce PM_2.5_ concentration in Xi’an and meet the national standard in winter, solving the problem of external diffusion is paramount. And this requires thorough national implementation of the ecological development strategy to improve the overall environmental quality. However, due to lack of accurate data on vehicle fuel consumption, this article failed to conduct a detailed analysis of a small area. In the future, a detailed analysis of a small area within a city will be added specifically to the effect of combustion theory on vehicle emissions.
